# Temperature effects on survival and dormancy patterns across age groups in vulnerable land snail *Vertigo moulinsiana*

**DOI:** 10.1007/s00114-026-02126-y

**Published:** 2026-06-16

**Authors:** Zofia Książkiewicz, Milena Wiśniewska, Bartłomiej Gołdyn

**Affiliations:** Department of General Zoology, Faculty of Biology, Adam Mickiewicz University, Uniwersytetu Poznańskiego 6, Poznań, 61–614 Poland

**Keywords:** Post-hibernation activation, Age classess, Climate change, Hibernation, Survival, Thermal stress

## Abstract

The Desmoulin’s whorl snail, *Vertigo moulinsiana* (Dupuy, 1849) (Gastropoda: Vertiginidae), an endangered micro-mollusc listed as Vulnerable in Annex II of the EU Habitats Directive, uses aestivation to survive environmental stress. This study aims to evaluate the effects of constant versus fluctuating thermal regimes on survival and activation dynamics of *V. moulinsiana.* Specimens from each age group individually (juveniles, subadults, adults distinguished based on the number of shell whorls and the presence of apertural barriers) were subjected to six thermal treatments: 23 °C, 35 °C, fluctuations between 25 °C and 35 °C, 4 °C, fluctuations between 4 °C and − 10 °C, and − 10 °C. Study shows that juveniles exhibited the lowest survival rates under low-temperature treatments, whereas adults displayed the tolerance to cold spells. Subadult survival was not significantly influenced by any temperature. Juveniles showed also higher survival under high‐temperature treatments. However, due to sensitivity to desiccation, these temperatures must occur with suitable air humidity. Importantly, temperature fluctuations substantially sped up activation time across all the age groups, whereas constant temperatures led to bet‐hedging response. Given the increasing frequency of extreme climate events under ongoing climate change, these results suggest even increased vulnerability of this species. Understanding its responses to environmental stress is therefore critical for getting to know protection strategies for *V. moulinsiana*.

## Introduction

Since the emergence of diverse animal phyla approximately 500 million years ago, Earth has experienced five major mass extinction events, each closely associated with significant and anomalous climatic changes (Kaiho 2025). Currently, we face an imminent sixth mass extinction, predominantly driven by anthropogenic factors that serve as the primary determinants of species declines (Lande 1998). Environmental stressors such as droughts and abrupt temperature fluctuations impose considerable thermal stress on animal populations, resulting in severe demographic impacts (e.g., Li et al. 2015). These effects are further exacerbated by habitat destruction and alteration, overexploitation, species translocations and introductions, as well as pollution. Collectively, these anthropogenic pressures elicit profound ecological and genetic consequences that elevate the risk of extinction (Ellis 2019; Davies et al. 2006).

Terrestrial land snails are among the animal groups most severely affected by extinction (Cowie et al. 2017). Their decline is driven by multiple anthropogenic factors, including habitat destruction, introduction of invasive species (Régnier et al. 2017), and climate change (Pokryszko 2003). Terrestrial gastropods are particularly sensitive to moisture deficits. Their population densities are closely linked to habitat physical characteristics such as humidity, shelter, and temperature – factors that often play a more critical role in determining their distribution than the availability of food resources (e.g., Boag 1985; Suominen 1999; Książkiewicz et al. 2013). As a result, alterations to habitats that affect microclimatic conditions can have profound impacts on terrestrial snail populations. Additionally, climatic disruptions, including rising annual temperatures and more frequent intense droughts, are expected to reduce snail activity and increase mortality rates (Luchtel and Deyrup-Olsen 2001).

The aim of this study was to evaluate the effects of significant temperature variations on survival and activation dynamics of the vulnerable micro-mollusc *Vertigo moulinsiana* (Dupuy, 1849). This species exhibits a globally declining population trend and is threatened across Europe, with climate change and habitat loss, being recognized as one of the primary drivers of its decline (Killeen et al. 2012). *Vertigo moulinsiana* is highly hygrophilous, inhabiting wetland environments, and is sensitive to temperature fluctuations (Książkiewicz-Parulska 2020). During summers and winters, the species remains attached to monocotyledonous plants, typically residing 30–50 cm above the ground or water surface level (Pokryszko 1990; Cameron 2003). Under unfavourable environmental conditions, it enters a state of dormancy (Killeen 2003). Such periods seem to be critical for the life history of the snail, since the organism is exposed to adverse environmental conditions and depends only on energy resources accumulated during the previous life stages. Thus, we conducted a series of laboratory experiments to compare mortality rates among three distinct age classes: juveniles, subadults, and adults of *V. moulinsiana* during dormancy under both constant temperature regimes and rapid temperature fluctuations. Additionally, we examined the influence of humidity changes on the termination of dormancy following these thermal expositions.

Given the known freeze tolerance of the species (Lipińska et al. 2025), we expected low‑temperature treatments to have relatively minor effects on survival. In contrast, we predicted that high‑temperature conditions would be the most detrimental, as *V. moulinsiana* is strongly hygrophilous and highly sensitive to desiccation. We further hypothesized that rapid thermal fluctuations would impose greater stress than constant high temperature, due to repeated transitions in metabolic state and increased evaporative water loss. We also expected juveniles to exhibit the highest mortality across treatments because of their smaller body size and limited energy reserves, with adults being the most resilient. Finally, we predicted that the snails kept in higher temperatures will activate faster, because they should absorb water faster and in lower temperatures their physiology should slow down.

## Materials and methods

Individuals of *Vertigo moulinsiana* used in the experiments were obtained from a captive breeding stock maintained under outdoor conditions (52.4672°N, 16.9249°E). The founder (P) generation originated from a natural population in north-western Poland (details in Książkiewicz-Parulska and Pawlak 2016), collected and breed under permit no. WPN-II.6401.269.2016.AG.2 issued by the Regional Directorate of Environmental Protection in Poznań, Poland. Experimental subjects were first-generation (F1) offspring. A total of 372 individuals were used in the study, comprising 72 adults (characterized by a fully developed shell with armature and lip), 132 subadults (shell with 3–3.5 whorls), and 168 juveniles (shell with 2 whorls).

### Experimental design

The experiment was designed to reflect both current and projected climatic conditions in the original locality of the studied snail population, representative of a broader region in Central-Eastern Europe. *V. moulinsiana* is a plant-dwelling species, typically found on monocotyledonous vegetation in wetland habitats, where air humidity remains high throughout most of the growing season. However, humidity can drop sharply - below 30% and even near zero. Such low-humidity conditions trigger dormancy in this hygrophilous species (Pokryszko 1990; Killeen 2003). Such declines under central European conditions usually occur during cold waves when temperatures fall well below freezing, periods of intense solar exposure, prolonged dry heatwaves (once rare, now increasingly frequent and persistent, Coffel 2025), or windy days (Wypych 2010; Zawadzka-Manko and Markowicz 2025).

All test tubes were prepared in the same way to maintain comparable conditions across all tubes. Snails were kept individually in a standard laboratory clear glass test tubes (6-ml, height: 100 mm; inner diameter: 9 mm). Consequently, the number of snails reported per treatment directly reflects the number of independent experimental replicates. The bottom of each tube was lined with a thin layer of water-saturated cotton wool to maintain humidity. The cotton layer was prepared as follows: dry cotton wool was placed in each tube, gently compressed to a thickness of approximately 4 mm, and moistened with 1 ml of water. Additionally, each individual was provided with a 100 mm dead leaf of sedge of similar thickness, collected from the litter layer in the natural habitat of *V. moulinsiana*, serving as a food source. The snails were subsequently placed in the tubes and gently misted with 1.5 ml of water per tube. All the vials were sealed with a dry cotton wool plug to allow ventilation while slowing down the desiccation.

Specimens were equally divided into six experimental groups, each consisting of 62 individuals: 12 adults, 22 subadults, and 28 juveniles. Each group was assigned to one of the temperature treatments described in a Table 1. The inclusion of different age classes allowed us to assess potential differences in thermal tolerance and activation dynamics, as different developmental stages may respond differently to environmental stress and may therefore represent critical or more vulnerable stages whose responses to environmental conditions can disproportionately influence population persistence. Thus, we aimed to identify the age class that may play the most important role in shaping population structure, particularly under temperature extremes associated with ongoing climate change.

**Table 1:** Experimental options – thermal conditions applied to snails during the seven-day experiment

Treatment	Abbreviation	Temperature exposition
Cool	C	Constant room temperature at 23 °C
Heat	H	Constant high temperature at 35 °C (corresponding to highest temperatures recorded in the field)
Heat waves	HW	Temperature fluctuations cycling between 25 °C (minimum) and 35 °C (maximum) every 12 h (fluctuations reflecting these typical for such events observed recently)
Constant low temperature	L	Constant low temperature at 4 °C, typical for the transition period of autumn or warm winter in natural conditions
Low temperature with transient freezing	LF	4 °C temperature with a 24-hour freezing period at -10 °C on day 4 (corresponding to cold snaps observed in the nature)
Constant freezing temperature	F	Constant freezing temperature at -10 °C (reflecting low winter temperatures)

Prior to exposure to experimental treatments, snails underwent a seven-day acclimatization period. During this time, they were kept individually in test tubes prepared in the same way as those used during the experiment. Before the start of the experiment, each snail was removed from the tube and stimulated with water to confirm that it was alive before being exposed to the experimental conditions. Individuals assigned to treatments C (constant room temperature), H (constant high temperature), and HW (heat waves) were maintained at room temperature conditions (23 °C), while those assigned to treatments L (constant low temperature), LF (low temperature with freezing), and CFT (constant freezing temperature) were placed under outdoor ambient conditions (autumn, under temperate climatic conditions typical for the region, with temperatures of approximately 10–14 °C during acclimatization). In all the cases the air humidity in the lab was between 45% and 55% and between 70% and 90% outdoor. At the end of acclimatization, the cotton wool substrate in all tubes was dry, and all snails remained inactive. Snails were considered active when the foot and tentacles were visibly extended outside the shell; individuals with the body fully retracted into the shell were classified as inactive.

During the experiment, target temperatures for cold treatments (L, LF, F) were maintained in temperature-controlled cold rooms. Snails in the room temperature control group (C) were kept in a climatized rearing room. For high-temperature treatments (H and HW), tubes with snails were held in water baths with temperature regulated by thermostatically controlled heaters. Fluctuations in the temperature (HW and LF) were set for 12 h to simulate the night-day temperature cycle observed in nature. All experimental snails were kept in complete darkness throughout the treatment period, with air humidity maintained at 40–50%. However, during the sub-zero treatment (LF, F), the humidity level dropped to 0.

Following seven days of the exposure to the experimental conditions, all tubes were transferred to laboratory room conditions (~ 23 °C, ~ 45% humidity) to assess the time required for snails to become active after the dormancy. We assumed that a change in temperature alone might be too weak a stimulus to terminate dormancy, and that in nature it may be combined with increased moisture (e.g., rain or fog). Thus, to separate the effects of temperature change from those of combined temperature and humidity changes on reactivation, half of the tubes in each treatment (with age classes equally represented) were gently sprinkled with boiled, cooled water, while the other half were not. In case of tubes maintained at constant room temperature (treatment C), all snails were sprinkled with water, as no temperature change occurred. These procedures were performed as rapidly as possible after removing from the experimental conditions, to minimize additional disturbance.

**Fig. 1:**
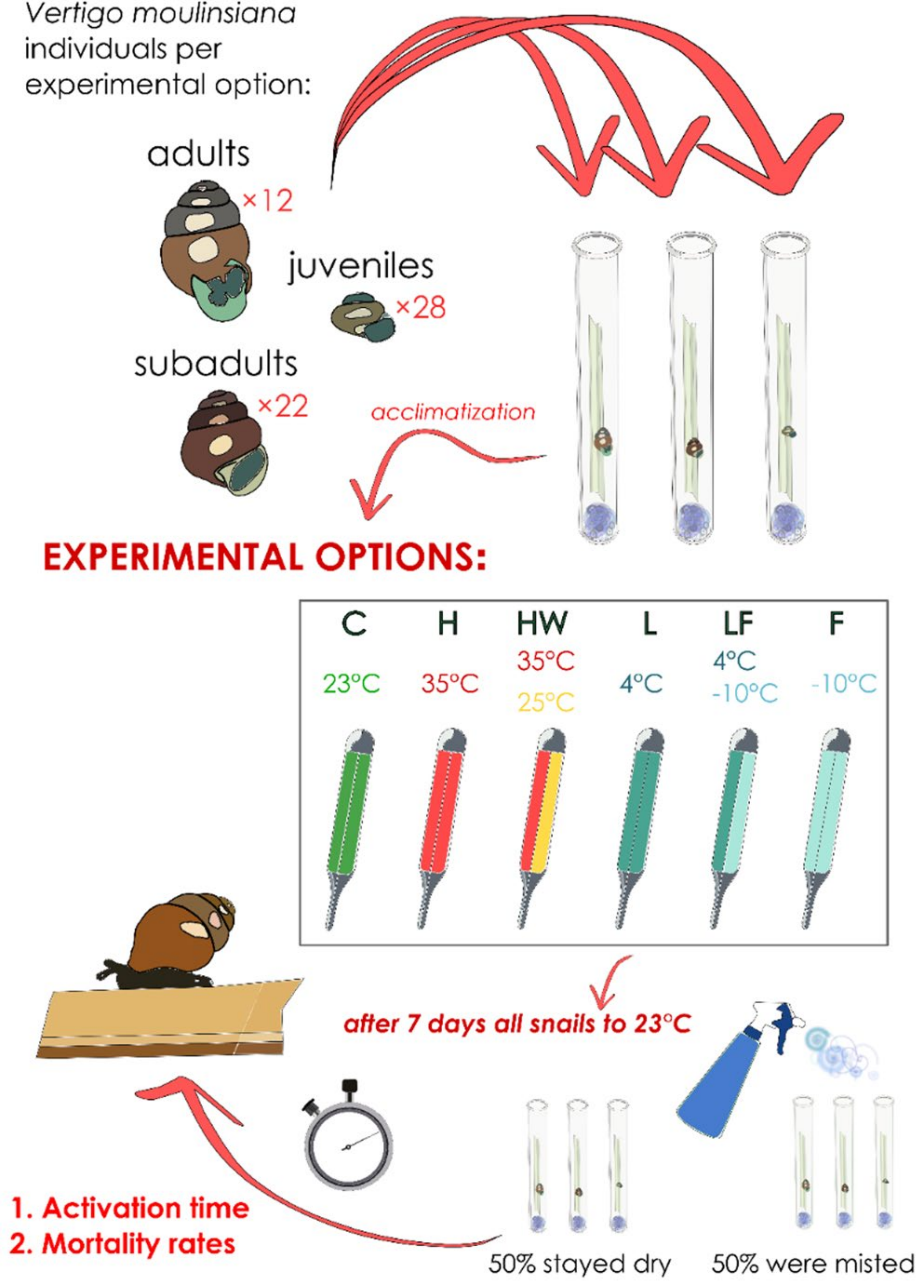
Experimental design. Six experimental groups of *Vertigo moulinsiana*, comprising adults (fully developed shells), subadults (shell with 3–3.5 whorls) and juveniles (shell with 2 whorls), were exposed to different thermal treatments: C, constant 23 °C; H, heat (constant 35 °C); HW, heat wave (alternating 35 °C and 25 °C every 12 h); L, constant low temperature (4 °C); LF, low temperature with freezing (4 °C with a 24 h period at -10 °C); and F, constant freezing temperature (-10 °C). Activation time and mortality rates were measured with and without misting

Snails were continuously observed for 12 h post-transfer, and the latency to full activity was recorded; full activity was defined as the eversion of both tentacles. If a snail remained inactive after 12 h, its position on the tube wall was marked with a marker, and checked for change in location after an additional 48 h. A lack of positional change during this period was taken as an indication of mortality Fig. 1.

### Statistical analyses

To compare activation times in the experimental treatments and between the age groups and check for the influence of increased humidity we used Cox Proportional-Hazards regression (Cox 1972; Therneau and Grambsch 2000). Time to full activation after treatment was introduced to the model as dependent variable while experimental treatment (six levels), age (three levels) and increased moisture (two levels) were the covariables. Six of the snails did not activate during the 12 h of observation following the treatment but were observed active after another 48 h. Since we have not got the exact activation time for them, these records were censored in the model. Data for the snails that were assumed dead after the experiment did not enter the Cox regression model, but we used them to test for the differences in survival rate between the age groups – that was performed using Chi-square test for each of the age groups and for all snails combined. To visualize the influence of tested factors on the activation time, forest plot was drawn based on univariate pairwise log-rank test for multiple comparisons of activation time (in seconds) within the levels of each of the three covariables (treatment, age, humidity) and the results were compared to the mean for all the experimental groups (Kassambara et al. 2024). Similar analysis, stratified for the age groups was applied to compare activation time (also in seconds) in each pair of the experimental treatments. Holm correction was applied on the resulting p-values, Kaplan-Meier Plot with cumulative number of activation events in each of the treatment groups was also used to illustrate the differences in activation dynamics between the experimental treatments. Calculations were performed in R version 4.2.3 (R Core Team 2023) under RStudio 2023.06.0 Build 421, using following packages: ‘survival’ (Therneau and Grambsch 2000; Therneau 2024), ‘survminer’ (Kassambara et al. 2024) and ‘survivalAnalysis’ (Wiesweg 2025). We considered p < 0.05 as a threshold determining statistical significance.

## Results

**Fig. 2:**
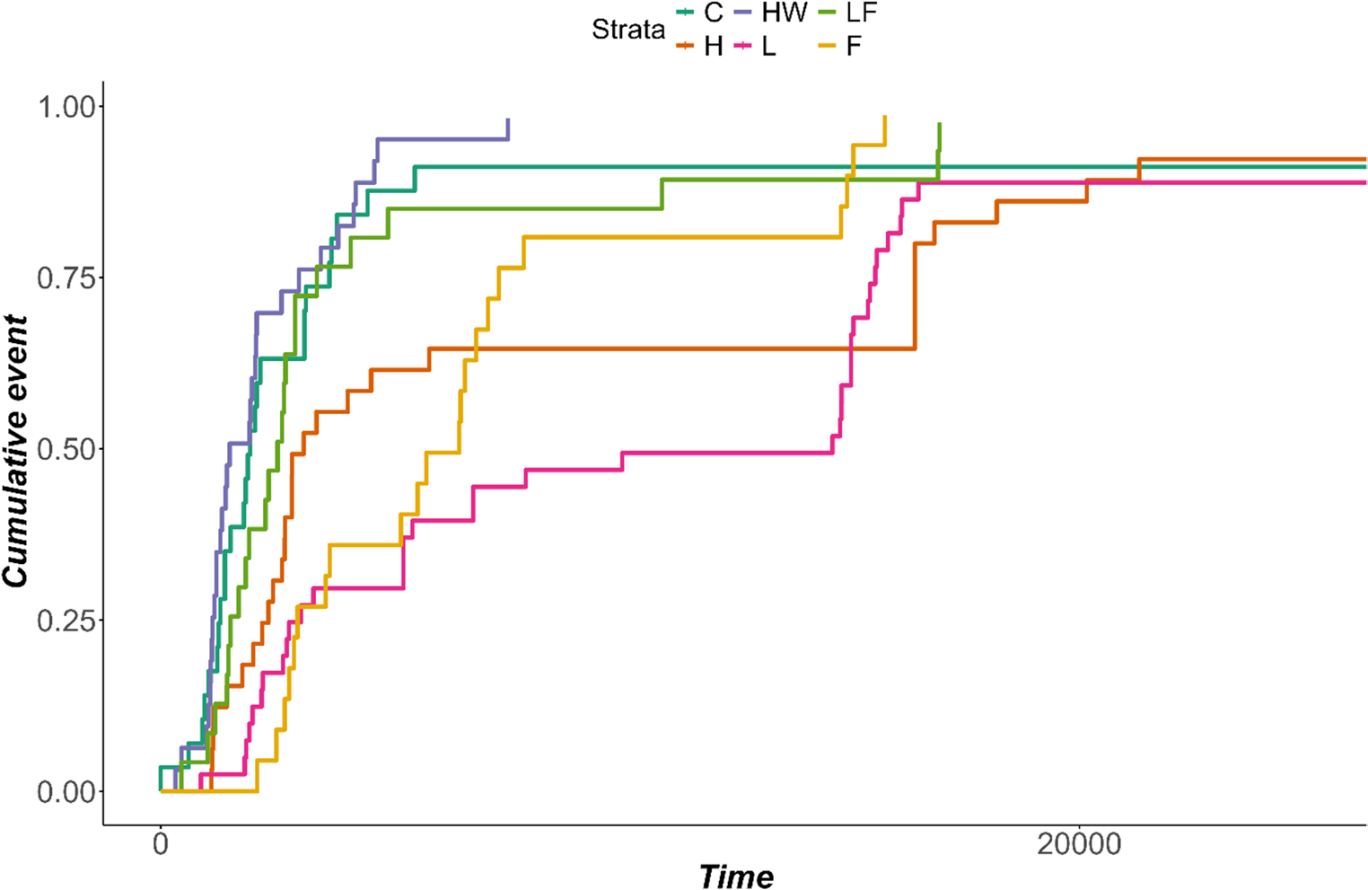
Kaplan-Meier Plot showing the differences in activation dynamics between the experimental treatments; C = constant room temperature (control), H = constant high temperature, HW = heat waves, L = constant low temperature, LF = low temperature with freezing, F = constant freezing temperature

There were significant differences in snail survival between the experimental treatments (Chi^2^ = 19.326, *p* = 0.0017), primarily due to differences in adult mortality (Chi^2^ = 23.333, *p* = 0.0003). Juveniles showed marginally significant differences in mortality between treatments (Chi^2^ = 11.38, *p* = 0.0444), whereas mortality of subadults did not differ significantly between the experimental treatments (Chi^2^ = 6.0662, *p* = 0.2998, Table 2., Fig. 2). As we hypothesized before the experiment, adult snails had the highest mortality rates in the constant room temperature (C) and heatwave (HW) treatments, and the lowest mortality in all low-temperature experiments (L, LF and F). In contrast, juveniles had the highest survival under warm temperature treatments (C and HW) and, as opposed to our initial expectations, in general, had lower mortality than adults.

**Table 2:** Mortality rates (in percent) in the analysed experimental treatments

	C	H	HW	L	LF	F
All snails	54.8	35.4	51.6	32.3	62.9	37.1
Juveniles	28.6	25.0	42.9	35.7	64.3	39.3
Subadults	68.2	45.5	50.0	40.9	63.6	40.9
Adults	91.7	41.7	75.0	8.3	58.3	25.0

**Fig. 3:**
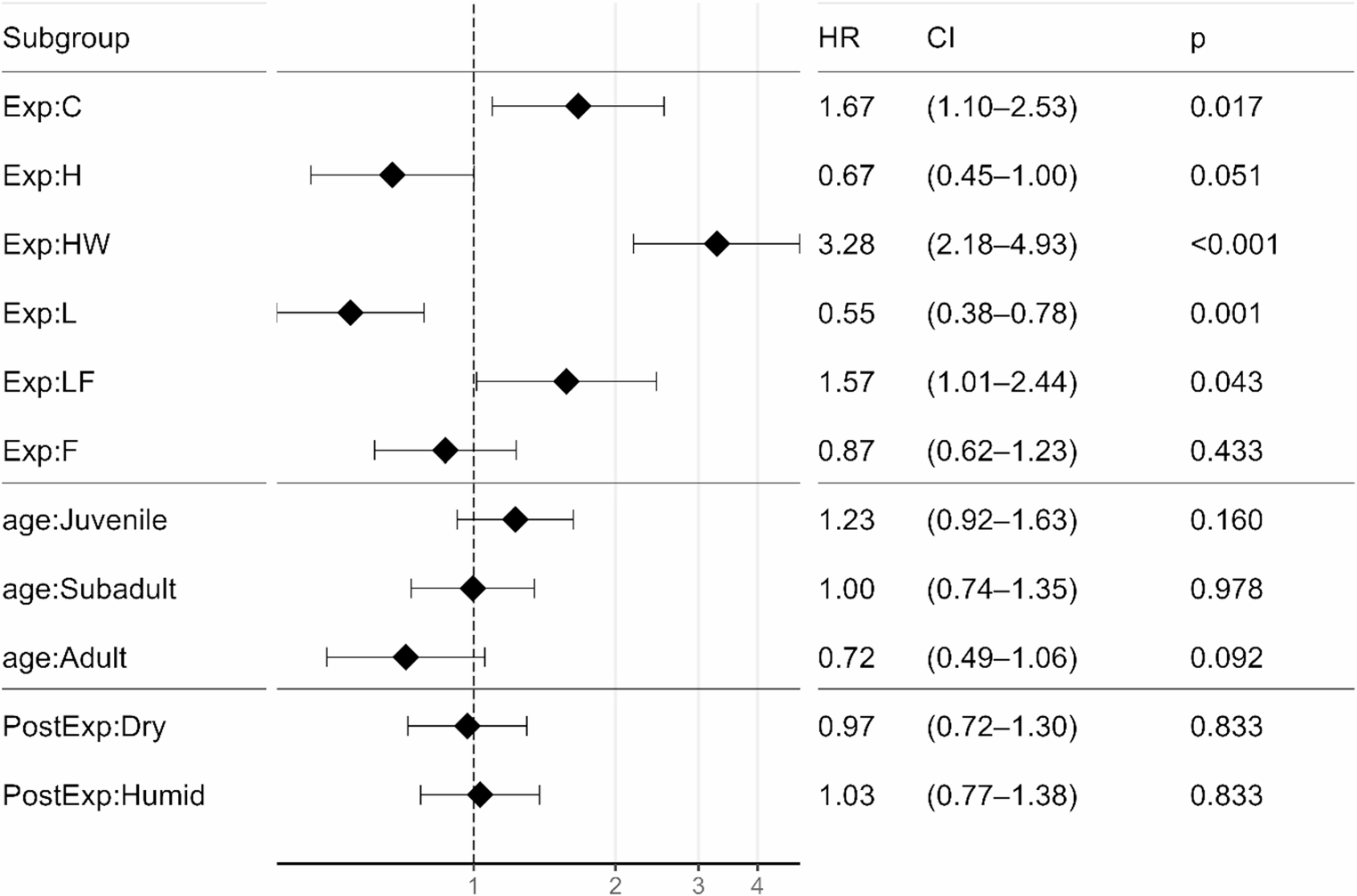
Forest plot showing the differences in activation time (HR) between particular levels of the three explanatory variables: experimental treatment (Exp), age (age) and increased humidity after the experimental dormancy (PostExp)

As a result, data on activation time were accessible for 100 juveniles, 66 subadult and 32 adult snails. Among the snails that survived the dormancy, those from the heat wave and room temperature treatments (HW and C) were the first to return to full activity, closely followed by snails kept at low temperature with periodic freezing (LF, Fig. 3). Activation dynamics were slowest in the constant low temperature treatment (L), followed by those kept at constant freezing temperatures (F) and in the hottest treatment (H). In these three treatments, two distinct groups of snails were visible as two separate steps on Kaplan-Meier Plot: the first group activated quickly (1000–3000 s) after the temperature was increased, while the second group delayed activation, terminating aestivation after a much longer period (approximately 6000-15,000 s).

According to Cox regression, the experimental treatment was the sole factor significantly influencing the dynamics of snail activation in our experiment (Chi^2^ = 46.5315, *p* < 0.0001). There were no such differences between the age groups of the snails (Chi^2^ = 2.5452, *p* = 0.280), and between vials with increased or normal humidity after the experiment (Chi^2^ = 0.7928, *p* = 0.373). Similar results gave the univariate log-rank analyses, comparing results for each experimental level to the average obtained from all the treatment: there were no significant differences between the age classes and the post experimental humidity level. Some of the experimental treatments, however, significantly differed from the average: constant low temperature (L) activated slower, whereas cool temperature (C), heat waves (HW), and low temperature with freezing (LF) activation time was faster than average. That was to some extent contrary to our predictions before the experiment, because we hypothesized that the snails will simply activate faster when kept in higher temperatures. Pairwise comparisons conducted between all the experimental treatments show that there were differences between the following pairs of treatments: control vs. constant low temperature; constant high temperature vs. heat waves; heat waves vs. constant low temperature, heat waves vs. constant freezing temperature and constant low temperature vs.

constant freezing temperature (Table 3).

**Table 3:** Results of pairwise comparisons between experimental treatments

	C	H	HW	L	LF	F
C		0.0723	0.1048	0.01344*	1	0.2616
H	-2.661		< 0.0001*	1	0.0723	1
HW	2.481	5.153*		< 0.0001*	0.1547	< 0.0001*
L	-3.258*	-0.488	-5.895*		0.01344*	0.5208
LF	0.084	2.686	-2.289	3.236*		0,2616
F	-1.983	0.901	-4.871*	1.513	-2.018	

upper diagonal: *p*-values resulting from multiple Cox regression analyses, with Holm’s correction, lower diagonal: corresponding z-values; asterisk – statistically significant differences

*C* constant room temperature, *H* constant high temperature, *HW* heat waves, *L* constant low temperature, *LF* low temperature with freezing, *F* constant freezing temperature

## Discussion

*Vertigo moulinsiana* is a unique snail species in temperate climates that aestivates and overwinters on monocot plants (Killeen 2003; Książkiewicz-Parulska et al. 2018), remaining directly exposed to fluctuating and sometimes sharply changing weather conditions. This study demonstrates that varying thermal conditions influence survival and activation dynamics differently among age classes of this vulnerable terrestrial snail, providing a new perspective to the factors underlying the unfavourable conservation status of this species.

### Age-dependent mortality rates under dormancy

Mortality rates differed between adults and juveniles across treatments, whereas subadults showed no significant differences in survival under different thermal conditions. Juveniles performed the worst under exposures involving low and freezing temperatures, likely due to sharp drops in humidity in the LF and F treatments, as subzero temperatures caused a marked decrease in air humidity. Juveniles are particularly susceptible to desiccation (Pokryszko 1990) because their higher surface-to-volume ratio (Killeen, 2003) accelerates water loss, and they lack apertural barriers that could reduce transpiration from the soft body (Pokryszko 1997).

Moreover, *Vertigo moulinsiana* is a freezing-avoidant species (Lipińska et al. 2025). This means that it survives low temperatures by avoiding internal ice formation, most likely through supercooling, a process in which body fluids are cooled below the freezing point without ice formation (Ansart and Vernon 2003). A reduction in total body water can further increase the concentration of cryoprotectants and decrease the proportion of freezable water (Ansart and Vernon 2003). Consequently, juveniles preparing for hibernation may be especially prone to desiccation, as they must reduce body water content to enhance supercooling capacity. Additionally, because juveniles allocate energy primarily to growth, they may invest less in accumulating energy reserves for overwintering. Some helicid snails, deposit polysaccharides (chiefly glycogen) in the digestive glands, which are depleted during hibernation (Bailey and Lazaridou-Dimitriadou 1991). There are no such data, however, for the juvenile snails.

In contrast, adults of *V. moulinsiana* tolerated low and freezing temperatures better than juveniles, but showed higher mortality under high-temperature treatments. This pattern may reflect differences in body size and surface-to-volume ratio: larger adults dissipate heat less efficiently than smaller juveniles, making them more vulnerable to overheating. For the same reason, juveniles may cope more effectively with high temperatures when humidity is maintained at a certain level.

### Dynamics of post-dormancy emergence

Experiments demonstrated that activation time depended solely on the thermal treatment, with no significant effect of age class or activation protocol (i.e. with or without water). This suggests that temperature is the key factor influencing activation after dormancy. Snails exposed to room temperature (23 °C; treatment C), heat-wave conditions (35 °C vs. 25 °C), and low-freezing conditions (4 °C vs. -10 °C) became active very quickly, within a comparable time frame across these treatments.

There is no single temperature threshold at which a particular snail species enters dormancy, and substantial interspecific variation has been reported (e.g. Kotsakiozi et al. 2012; Cameron 2016). However, based on the available literature, a temperature of around 23 °C does not induce dormancy, thus in the case of our treatment low humidity was probably the only reason why the snails entered this state. In contrast, when temperatures drop below approximately 7 °C, snails typically begin hibernation (Ștef et al. 2025), whereas exposure to 30 °C often proves lethal (Cameron 2016), making aestivation essential for survival. The relatively rapid activation observed after exposure to 23 °C may therefore simply indicate that the snails entered only a shallow dormant state, which was easily interrupted by changes in external conditions, such as an increase in humidity.

Temperature fluctuations – such as those imposed in the heatwave (HW) and cold-spell (LF) treatments – appear insufficient to induce deep dormancy in *Vertigo moulinsiana*. Instead, they seem to stimulate the maintenance of low-level physiological activity, keeping individuals in a metabolically responsive state until favourable conditions return. In this species, individuals have been shown to emerge and remain active even at temperatures as low as 0–2 °C (Lipińska et al. 2025). Thus, fluctuating regimes provide no consistent cue to enter deep dormancy but instead sustain a general metabolic readiness. In contrast, stable extreme thermal conditions, either low or high (in our experiments, 4 °C and 32 °C, respectively), appear to facilitate entry into deep dormancy.

A relatively quick emergence from dormancy under fluctuating conditions may be advantageous, allowing snails to exploit short-term opportunities for feeding or activity whenever suitable conditions arise. Interestingly, among snails exposed to constant low or high temperatures, we observed two distinct “wake-up” waves: the first group became active within less than an hour, while the second group, clearly separated in time, reactivated after 1.5 h or more. This pattern may suggest a bet-hedging strategy (Ripa et al., 2010), reflecting an adaptation to unpredictable environments, where relying on a single behavioural response could increase the risk of offspring loss.

Nevertheless, the absence of significant age‑related differences should be interpreted with caution. Because adult mortality was high, the number of individuals contributing activation‑time data in this group was much smaller than for juveniles and subadults (32 vs. 100 and 66 individuals, respectively). This imbalance may have reduced the statistical power of our analyses, increasing the likelihood of a Type II error and masking a real age effect. Future experiments with more evenly represented age classes would help resolve this limitation and allow a more reliable assessment of age‑specific responses.

## Conclusions

Land snails, as poikilothermic organisms, are strictly dependent on temperature fluctuations and must continuously adjust to ambient conditions to survive unfavourable hot and cold periods. Our findings indicate that temperature influences survival across age groups and affects dormancy and reactivation in *Vertigo moulinsiana* under laboratory conditions. The species’ responsiveness to fluctuating conditions may allow rapid exploitation of short favourable periods. However, this same sensitivity may increase vulnerability to the rising frequency and intensity of temperature extremes expected under climate change.

## Data Availability

No datasets were generated or analysed during the current study.
